# Supplementary material for: Computational principles of neural adaptation for binaural signal integration
Source: PLoS Comput Biol. 2020 Jul 17;16(7):e1008020. doi: 10.1371/journal.pcbi.1008020 (PMC7398554; doi:10.1371/journal.pcbi.1008020)
Supplement: S1 Text — (PDF) [file pcbi.1008020.s001.pdf]

S1 Text. ILD Computation. For calculating the ILD values of real world stimuli we use the text book definition (e.g [1]) of ILD computation:

$$ILD = 10 * log_{10} \frac{\int_{t=0}^{\infty} s_l^2(t)}{\int_{t=0}^{\infty} s_r^2(t)} \tag{1}$$

where s_l is the signal received at the left ear and s_r is the signal received and the right ear at time t.

References

[1] Irvine DR. Physiology of the auditory brainstem. In: The mammalian auditory pathway: Neurophysiology. Springer; 1992. p. 153–231.
