# Supplementary material for: Computational principles of neural adaptation for binaural signal integration
Source: PLoS Comput Biol. 2020 Jul 17;16(7):e1008020. doi: 10.1371/journal.pcbi.1008020 (PMC7398554; doi:10.1371/journal.pcbi.1008020)
Supplement: S2 Text — (PDF) [file pcbi.1008020.s002.pdf]

S2 Text Physiological neuron parameters.

The purpose of the presented model architecture is to capture dynamics and adaptation of the system states to yield insights on neural circuitry and functionality. However, to present the model in a physiologically more realistic setting and to provide testable neurophysiological parameter values, we modified Eq. (1) and Eq. (6) by incorporating membrane capacitance C and leak reversal potential E_l. The core equations read

$$\frac{1}{\tau}\cdot C\cdot\frac{dV_r}{dt} = g_l\cdot(E_l-V_r)+(\beta-V_r)\cdot\sum_{\omega'} s^r_{\omega'}\bar{\Lambda}^{E-E}_{\omega\omega'}-(\gamma-\kappa*(E_l-V_r))\cdot\sum_{\omega'} g_q(q_{\omega'})\bar{\Lambda}^{E-I}_{\omega\omega'} \tag{1}$$

for LSO neurons and

$$\frac{1}{\tau}\cdot C\cdot\frac{dV_q}{dt} = g_l\cdot(E_l-V_q)+(\beta-V_q)\sum_{\omega'} s^q_{\omega'}\Lambda^{I-E}_{\omega\omega'} \tag{2}$$

for MNTB neurons, respectively. Here, τ is the membrane time constant and V_r and V_q the membrane voltage of LSO and MNBT model neurons, respectively. With these equations, physiological neuron parameters are derived. Values for these parameters are chosen according to neurophysiological experiments [1, 2, 3] and are in line with other models of LSO neurons (see [4]). Parameter γ_r and κ_r are chosen to fit default responses shown in Fig. 2:

Table 1: Model parameters

Neuron Model			
τ	0.25 ms	g_l	26.4 nS
E_l	-60 mV	C	24 pF
γ_r	$4\cdot10^{-8}$	κ_r	$12\cdot10^{-7}$

Applying these model equations we repeat experiment 1 and demonstrate that the response behavior is similar to our model notation (see S5 Fig B). Model inputs are now in range $0-500pA$ (see S5 Fig A). Influence of parameter values $gamma_r$ and $kappa_r$ (see S5 Fig C,D) are similar to our model notation.

References

[1] Sanes DH. An in Vitro Analysis of Sound Localization Mechanisms in the Gerbil Lateral Superior Olive. Journal of Neuroscience. 1990;10(11):3494–3506. doi:10.1523/JNEUROSCI.10-11-03494.1990.

[2] Adam T, Finlayson P, Schwarz D. Membrane properties of principal neurons of the lateral superior olive. Journal of neurophysiology. 2001;86(2):922–934.

[3] Barnes-Davies M, Barker MC, Osmani F, Forsythe ID. Kv1 currents mediate a gradient of principal neuron excitability across the tonotopic axis in the rat lateral superior olive. European Journal of Neuroscience. 2004;19(2):325–333.

[4] Ashida G, Tollin DJ, Kretzberg J. Physiological models of the lateral superior olive. PLOS Computational Biology. 2017;13(12):1–50. doi:10.1371/journal.pcbi.1005903.
