# Supplementary material for: Computational principles of neural adaptation for binaural signal integration
Source: PLoS Comput Biol. 2020 Jul 17;16(7):e1008020. doi: 10.1371/journal.pcbi.1008020 (PMC7398554; doi:10.1371/journal.pcbi.1008020)
Supplement: S3 Text — (PDF) [file pcbi.1008020.s003.pdf]

S3 Text Influence of noise on coding precision.

The standard separation index [1] between two stimuli is calculated by dividing the difference in firing rates by the geometric mean of the standard deviation of their firing rates over trials. The presented model has no internal noise source, thus fixed inputs will result in identical model responses. Trial-to-trial variability can be achieved by adding noise to model inputs. Here, we demonstrate that adding noise to model inputs does not substantially influence the coding precision values when repeated measurements are taken, i.e. over trials our coding precision index is similar to the standard separation (see S6 Fig). The difference in the graphs results from the calculated standard deviation values. However, these values do not depend on the model itself but on the standard deviation of the incorporated noise source. The noise source is Gaussian white noise with $\sigma = 0.05$ and mean continuously centered on the input value, that is $s_\omega^{\{r,q\}}(t) = \mathcal{N}(s_\omega^{\{r,q\}}(t), \sigma)$.

To demonstrate that noisy inputs do not substantially change the results of the adaptation and timing experiment (3,4), we regenerate Fig. 5 for noisy inputs and averaged the responses over trials (25 for timing experiment and 100 for adaptation experiment). For each measurement we calculate the standard separation index (see S7 Fig).

We introduced the coding precision index to calculate the difference in response between two stimuli without considering a noise model. From the results generated with the model that is extended with a noise source we conclude that the noiseless index calculation does not substantially differ from the separation index and is sufficient to provide a means to quantify the coding precision of model neurons. The marginal differences in the adaptation values (percentage and dB) result from the noisy inputs and do not change the explanatory power of the conducted experiments.

References

[1] Sakitt B. Indices of Discriminability. Nature. 1973;241(5385):133–134. doi:10.1038/241133a0.
