# Supplementary material for: Computational principles of neural adaptation for binaural signal integration
Source: PLoS Comput Biol. 2020 Jul 17;16(7):e1008020. doi: 10.1371/journal.pcbi.1008020 (PMC7398554; doi:10.1371/journal.pcbi.1008020)
Supplement: S1 Fig — (PDF) [file pcbi.1008020.s005.pdf]

S1 Fig. Parameter influence on coding precision.

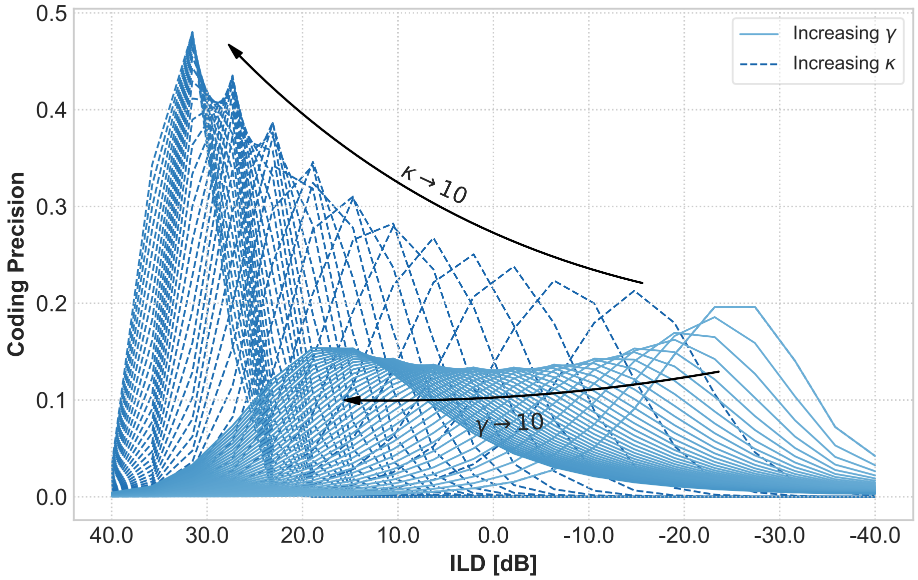

Parameter influence on coding precision. Dashed blue lines indicate the coding precision of the model response for changing κ_r parameter. For increasing parameter value the coding precision increases while simultaneously the response range decreases (the ILDs that the model exhibits a response). Solid blue lines indicate the coding precision of the model response for changing γ_r parameter. For increasing parameter value the coding precision and extensions of the response range remains almost constant. However the response range itself shifts from negative to positive ILD values. A proper ratio of the two parameters facilitates a balance between high coding precision and response range.
