# Supplementary material for: Computational principles of neural adaptation for binaural signal integration
Source: PLoS Comput Biol. 2020 Jul 17;16(7):e1008020. doi: 10.1371/journal.pcbi.1008020 (PMC7398554; doi:10.1371/journal.pcbi.1008020)
Supplement: S3 Fig — (PDF) [file pcbi.1008020.s007.pdf]

S3 Fig. Time-intensity trading experiment Park et al. 1996.

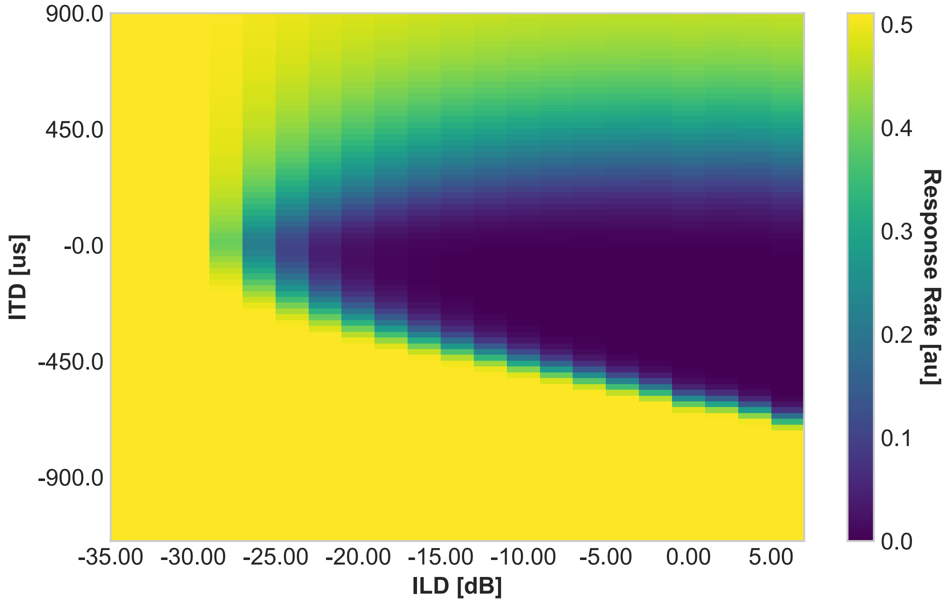

Time-intensity trading of LSO neurons. Figure shows simulation result for time-intensity trading experiment similar to [1]. To achieve similar results the model parameters needed to be changed to $\gamma = 10$ and $\kappa = 0$. This change of parameters is reasonable, since the effectiveness of the inhibitory inputs might differ from neuron to neuron [2, 3]. The ipsilateral stimulus intensity was fixed to $35dB$, whereas the contralateral input intensity varied between $0dB$ and $40dB$ thus creating similar ILD values as in the experiment of [1]. The duration of a single stimulus was set to 300 µs. To achieve qualitatively similar results as the authors (compare their Fig. 3) we choose the time-intensity value of the arrival times of the model inputs to be 10 µs/dB. We use this value for the timing experiment (no. 4) to adapt the arrival time of inputs.

References

[1] Park TJ, Grothe B, Pollak GD, Schuller G, Koch U. Neural Delays Shape Selectivity to Interaural Intensity Differences in the Lateral Superior Olive. Journal of Neuroscience. 1996;16(20):6554–6566. doi:10.1523/JNEUROSCI.16-20-06554.1996.

[2] Reed MC, Blum JJ. A model for the computation and encoding of azimuthal information by the lateral superior olive. The Journal of the Acoustical Society of America. 1990;88(3):1442–1453.

[3] Park TJ, Monsivais P, Pollak GD. Processing of Interaural Intensity Differences in the LSO: Role of Interaural Threshold Differences. Journal of Neurophysiology. 1997;77(6):2863–2878. doi:10.1152/jn.1997.77.6.2863.
