# Supplementary material for: Computational principles of neural adaptation for binaural signal integration
Source: PLoS Comput Biol. 2020 Jul 17;16(7):e1008020. doi: 10.1371/journal.pcbi.1008020 (PMC7398554; doi:10.1371/journal.pcbi.1008020)
Supplement: S4 Fig — (PDF) [file pcbi.1008020.s008.pdf]

S4 Fig. Adaptation to lower adapter tone levels.

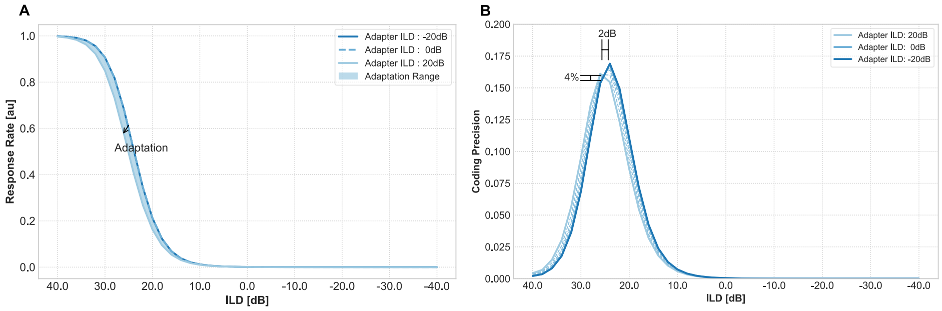

Adaptation to lower adapter tone levels. Same stimulus paradigm as in experiment no. 3 (Fig. 4**B**) except that the adaptor tone intensity was limited to range $-20dB$ and $+20dB$, which is similar to the highest ILD value used in [1]. (A) the maximum adaptation range decreases and so does the coding precision value (B) which creates qualitatively similar results to then one Gleiss and colleagues found ([1] compare their Fig. 4).

References

[1] Gleiss H, Encke J, Lingner A, Jennings TR, Brosel S, Kunz L, et al. Co-operative population coding facilitates efficient sound-source separability by adaptation to input statistics. PLoS biology. 2019;17(7):e3000150.
