# Supplementary material for: Computational principles of neural adaptation for binaural signal integration
Source: PLoS Comput Biol. 2020 Jul 17;16(7):e1008020. doi: 10.1371/journal.pcbi.1008020 (PMC7398554; doi:10.1371/journal.pcbi.1008020)
Supplement: S5 Fig — (PDF) [file pcbi.1008020.s009.pdf]

S5 Fig. Model behavior for physiological neuron parameters.

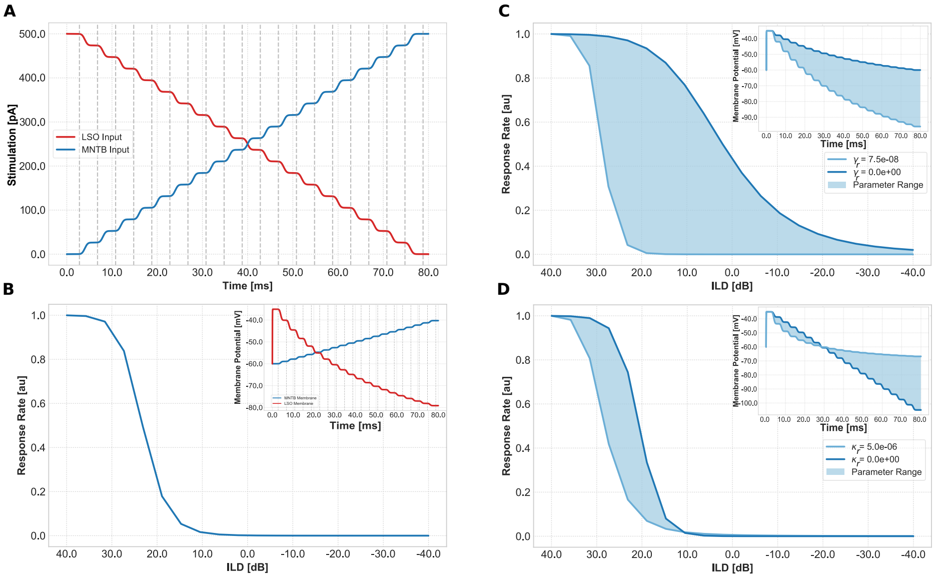

Model behavior for physiological neuron parameters. (A) Current input to model neuron in range 0 to 500pA. Red line shows excitatory input from cells of the ipsilateral cochlear nucleus to LSO neurons. Blue line shows excitatory input from cells of the contralateral cochlear nucleus to MNTB neurons which are converted to inhibitory input to LSO neurons. Vertical grey dashed lines indicate measurement points of neuron's response. (B) Model response of an LSO neuron for a chosen set of parameter values. Blue line shows the response rate of a single LSO neuron to stimuli as presented in (A) over interaural level difference. Inset shows membrane potentials in mV for LSO (red) and MNTB (blue) neurons. (C,D) Model response for parameter values γ_r and κ_r, respectively. (C) Parameter values of γ_r are varied in range $[0.0, 7.5 \cdot 10^{-8}]$ which control the strength of subtractive inhibition. (D) Model responses for different κ_r values in range $[0.0, 5.0 \cdot 10^{-6}]$ that regulates the effect of shunting inhibition on the membrane potential. Filled areas are approximated from simulation results.
