# Supplementary material for: Computational principles of neural adaptation for binaural signal integration
Source: PLoS Comput Biol. 2020 Jul 17;16(7):e1008020. doi: 10.1371/journal.pcbi.1008020 (PMC7398554; doi:10.1371/journal.pcbi.1008020)
Supplement: S6 Fig — (PDF) [file pcbi.1008020.s010.pdf]

S6 Fig. Influence of noise on coding precision.

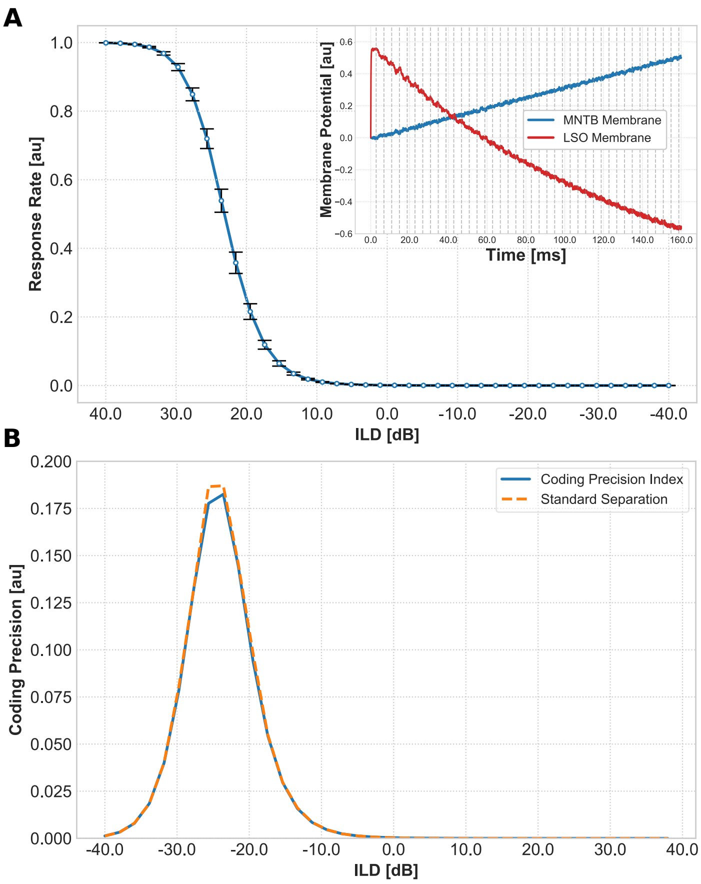

Influence of noise on coding precision. (A) Mean model response over 250 trials (blue line). Black bars indicate standard deviation for each measuring point. Inset shows noise influence on membrane potential over time. (B) Coding precision index (solid blue line, difference between neighboring measurements from (A)) and the standard separation index (dotted Orange line, difference between neighboring measurements from (A) divided by the geometric mean of their standard deviations) is shown. The difference between the two curves neglectable.
