# Supplementary material for: Computational principles of neural adaptation for binaural signal integration
Source: PLoS Comput Biol. 2020 Jul 17;16(7):e1008020. doi: 10.1371/journal.pcbi.1008020 (PMC7398554; doi:10.1371/journal.pcbi.1008020)
Supplement: S7 Fig — (PDF) [file pcbi.1008020.s011.pdf]

S7 Fig. Standard separation index.

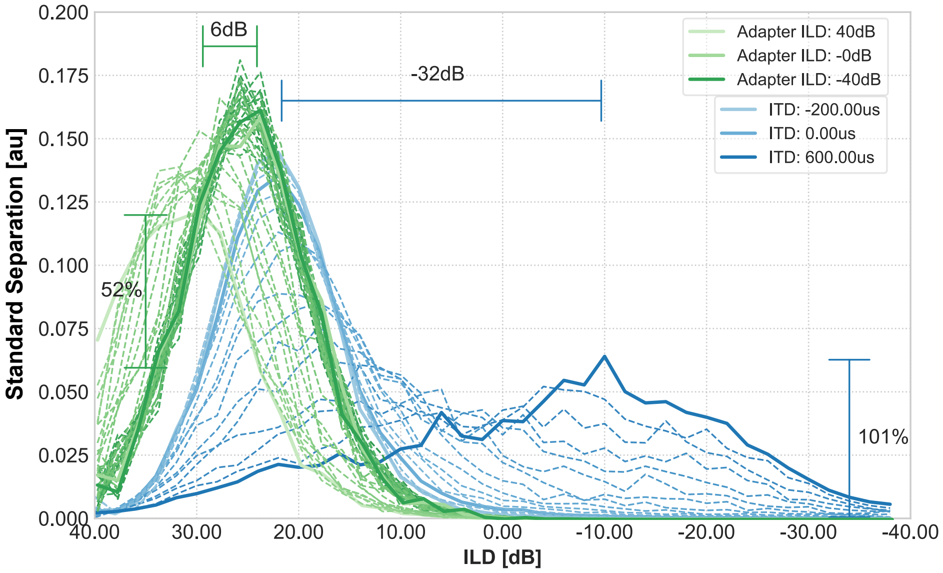

Standard separation index. Standard separation index for experiment 3, adapter tone experiment (green lines) and 4, timing experiment (blue lines). The peak of the green curve is shifted by a maximum of $6dB$ for increased adapter tone intensity and gains an sensitivity increment of 52%, compared to $6dB$ shift and sensitivity increment of 53% for no noise inputs (compare Fig. 5). The peak of the blue curve is shifted by a maximum of $32dB$, compared to $38dB$ between stimuli of different ITDs. The increment of sensitivity for this shift is 101%, compared to 106%.
